# Prevalence and risk factors of diabetes and impaired fasting glucose in Nauru

**DOI:** 10.1186/1471-2458-11-719

**Published:** 2011-09-23

**Authors:** Amina Khambalia, Philayrath Phongsavan, Ben J Smith, Kieren Keke, Li Dan, Andrew Fitzhardinge, Adrian E Bauman

**Affiliations:** 1 Clinical and Population Perinatal Research, Kolling Institute of Medical Research, University of Sydney, Uni Dept O&G, Building 52, Royal North Hospital, St Leonards NSW 2065, Australia; 2 Sydney School of Public Health, University of Sydney, Sydney, NSW 2006, Australia; 3 School of Public Health and Preventive Medicine, Monash University, PO Box 197, Caulfield East, Victoria 3145, Australia; 4 Government of Nauru, Republic of Nauru; 5 World Health Organization Office for the South Pacific/Division of Pacific Technical Support, Suva, Fiji; 6 Biochemistry Dept-GDU, Concord Repatriation General Hospital, Concord, NSW 2137, Australia

**Keywords:** risk factors, epidemiology, adult diabetes, population studies

## Abstract

**Background:**

No comprehensive assessment of diabetes prevalence in Nauru has been conducted since an extreme prevalence was documented more than two decades ago. This study aims to determine the prevalence and risk factors of diabetes and impaired fasting glucose.

**Methods:**

A nationwide survey in 2004 of people aged 15- 64 years (n = 1592). Fasting plasma glucose levels were used to defined diabetes (≥7.0 mmol/l or 126 mg/dl) and prediabetes (6.1-6.9 mmol/l or 110-125 mg/dl).

**Results:**

The sex-standardized prevalence of diabetes was 13.0% (95% CI: 10.6, 15.4) in men, 14.4% (11.9, 16.9) in women, and 13.7% (12.0, 15.4) combined. The sex-standardized prevalence of prediabetes was 6.4% (4.6, 8.2) for men, 5.5% (3.9, 7.2) for women, and 6.0% (4.8, 7.3) combined. The prevalence of diabetes for individuals 15-24, 25-34, 35-44, 45-54 and 55-64 years was 4.5%, 7.6%, 24.1%, 32.9%, and 42.7%, respectively. The prevalence of prediabetes for the same age categories was 4.2%, 8.8%, 5.9%, 6.6%, 7.1%, respectively. Multivariable, multinomial logit modeling found risk factors for prediabetes were high cholesterol levels (OR: 2.02, 95% CI: 1.66, 2.47) and elevated waist circumference (OR: 1.04, 95% CI: 1.00, 1.08), and for diabetes were age in years (OR: 1.06; 95% CI: 1.04, 1.07), cholesterol levels (OR: 1.84, 95% CI: 1.58, 2.14) and waist circumference (OR: 1.04, 95% CI: 1.02, 1.07).

**Conclusions:**

Diabetes remains a major public health problem in Nauru, affecting one out of every ten people. While the prevalence of diabetes has declined, its burden has persisted among the old but also extended towards the younger age groups.

## Background

The prevalence of diabetes in Nauru has previously been reported to be extremely high. In 1975, the prevalence of diabetes in Nauru was 34.4%, ranking Nauruans second in the world for highest ever recorded rate of diabetes [1]. The highest rate of diabetes ever recorded in the scientific literature is among the American Pima Indians [2]. A 1965 study among American Pima Indians aged 35 years and older found age-and sex-specific prevalence rates ranging from 34.2% to 68.3% [2]. Surveys in Nauru in 1976 and in 1982 found similarly high rates of diabetes, with prevalence estimates of 29.0%[3], and 23.9% [4], respectively. On an age-standardized basis, the incidence of diabetes in Pima Indians was 38.2 cases/1,000 person-years in 1965, compared to 26.2 cases/1,000 person-years in1975/1976-1982 among Nauruans [2,5].

Explanations for the epidemic proportions of diabetes, heart disease, obesity and hypertension in Nauru include: urbanization, low physical activity, the importation of processed foods and a diabetic genotype [3,6]. After independence in 1968, Nauru became the smallest and richest republic (per capita) in the world by exploiting large deposits of phosphate [3]. During a period of excessive wealth, the indigenous population in Nauru, which is predominantly of Micronesian ancestry, became more dependent on store-bought food and accustomed to a sedentary lifestyle [6].

The prevalence of diabetes for all age-groups worldwide estimated to be 2.8% in 2000 is projected to increase to 4.4% by 2030 [7]. However, Nauru may be one of the few exceptions in the world where diabetes prevalence may actually decrease. A trend analysis of the prevalence of non-insulin-dependent diabetes mellitus (NIDDM) and impaired glucose tolerance (IGT) among Nauruans aged 20 years and older showed the age-standardized prevalence (95% CI) of NIDDM decreased from 27.9% (23.8, 32.0) in 1975/6 to 24.7% (22.7, 26.6) in 1982 and then to 24.0% (22.1, 25.9) in 1987 [5]. The age-standardized prevalence of IGT fell by more than half from 1982 to 1987 [5]. Results indicate that there was a decline in the incidence of IGT and NIDDM in subjects with normal glucose at baseline [5]. The study found no changes in the frequency of recognized risk factors (physical activity, cigarette smoking, obesity, triglycerides or cholesterol), leading the authors to postulate that a high proportion of genetically susceptible individuals with the 'thrifty genotype' had been removed from the pool [5]. Since these analyses were conducted almost two decades ago there has been no further information on the prevalence and risk factors of diabetes in Nauru.

As part of a global effort to address the increasing burden of chronic diseases, the Government of Nauru and the World Health Organization in 2004 implemented a nation-wide Stepwise Surveillance of Risk Factors for Non-communicable Diseases Survey (Nauru-STEPS) to document the patterns and levels of diabetes and its associated risk factors [8]. This paper describes the age-specific and sex-standardized prevalence of diabetes and impaired fasting glycemia (IFG) in a randomly selected and nationally representative sample of people ages 15-64 years in Nauru and examines the associations between diabetes and a range of behavioral and biochemical risk factors.

## Methods

### Study setting and design

Nauru is situated in the Central Pacific Ocean. Only 21 square kilometers (8.1 square miles) in size, Nauru is one of the smallest nations in the world. The country consists of one main island, divided into 14 small districts. Indigenous Nauruans, Kiribati and Tuvaluan residents comprise approximately 90% of the total population of around 11,000 [9], and were included in the survey. Expatriate residents were excluded, as well as individuals with mental illness, physical or developmental disabilities.

Data from the Birth, Deaths, Marriages Register, Hospital Medical Record Register, 2003 Electoral Roll, and Nauru Phosphate Corporate database were used to create a sampling frame. A simple random sampling technique, stratified by age and sex was then employed. Survey staff visited the randomly selected participants at their homes to invite them to participate and to attend the health clinic for interviews, biochemical and physical measurements. Data collection occurred from June to October 2004. Detailed survey methods have been published elsewhere [8].

The survey was a collaborative initiative between the Nauru Ministry of Health, the World Health Organization and the Centre for Physical Activity and Health at the University of Sydney in Australia. Ethics approval was granted by the University of Sydney Human Research Ethics Committee and the Nauru Non-communicable Disease Steering Committee, Nauru Ministry of Health.

### Data collection

#### Behavioral risk factors

Information on behavioral risk factors was collected from consenting participants using a face-to-face structured interview which included questions on smoking, alcohol consumption, fruit and vegetable consumption, and physical activity [8]. Interviews were conducted in English, Nauruan, Kiribati or Tuvaluan by staff trained in the interview protocol, and fluent in the local vernacular. Interviews were conducted in private and ranged from 30-50 minutes in duration.

#### Anthropometry measurements

Trained survey personnel performed height, weight, blood pressure and waist circumference measurements. Height was measured once with a Portable Height Scale to the nearest 0.1 cm. Weight was measured once to the nearest 0.1 kg with the Seca Scale. Height and weight measurements were taken in light clothing, with shoes, socks, and head gear removed. Waist circumference was measured once to the nearest 0.1 cm with the Figure Finder constant tension tape. Blood pressure (BP) and heart rates were measured with the Omron T5 Automatic Blood Pressure Monitor. Both BP and heart rates were measured three times, and the mean value of the second and third readings was used in the analysis. Quality control for each measurement was monitored through periodic checks conducted by the survey supervisor.

#### Laboratory analysis

A whole blood sample was collected into separate vacutainer collection tubes for blood lipid and glucose analyses. Venous EDTA plasma blood lipids and glucose were tested on site within 2 hours of collection using a Reflotron biochemical analyzer (Roche Reflotron Meter). When presenting at the blood collection laboratory, all participants were asked about their fasting status. Participants who had not fasted for at least 8 hours were asked to return the next day after fasting. Details on the laboratory analysis have been published elsewhere [8].

#### Definitions of diabetes

Diabetes was defined as a fasting plasma glucose value ≥7.0 mmol/l (126 mg/dl) or a previous diagnosis of diabetes [10]. Diabetes was further sub classified as "known diabetes" if diabetes had been diagnosed previously by a medical practitioner or as "newly diagnosed diabetes" if diabetes was first diagnosed by this study. Prediabetes was defined as a fasting plasma glucose value of 6.1-6.9 mmol/l (110-125 mg/dl) in accordance with WHO criteria [10]. Participants not fulfilling these criteria were considered to have normal glucose tolerance.

#### Statistical Analysis

Descriptive statistics were computed as unadjusted means (± SD) and compared using Student's *t *test for continuous variables and *X*^2 ^test for categorical variables. Age-specific and sex-standardized prevalence and the 95% confidence intervals (CIs) were calculated against the 2002 Nauru census data [9]. Data were weighted in all analyses. Weighting adjusted for age and sex. Post-stratification weighting was computed for Nauru-STEPS to bring the sample data to the 2002 Census estimates of the Nauruan population aged 15-64 years and to adjust for certain age/sex stratum being over- or under-represented in the survey data [8].

A multinomial logit analysis was used to examine the association between demographic, lifestyle and metabolic factors and the odds of diabetes and prediabetes. The independent variables used in the multivariable analysis included: age, sex, years of education, body mass index (BMI), systolic blood pressure, diastolic blood pressure, total cholesterol, alcohol risk category, smoking status, physical activity at work, physical activity during leisure time and a family history of diabetes. Reported *P *values were two tailed and *P *≤ 0.05 was considered to be statistically significant. Standardized rates were computed by the direct method using the sex- and age-specific distribution of the national census in 2002 from the Nauru National Bureau of Statistics Office. Statistical analysis was performed using SAS for Windows version 9.2 (SAS Institute Inc, Carey, North Carolina). Age-adjusted means ± SEM was performed using STATA version 11.0 complex survey commands.

## Results

The Nauru-STEPS survey included a representative sample of 2, 272 participants aged 15-64 years (response rate = 89.7%), which represents 15-20% of the total population of Nauru.^9 ^Cases with irregular fasting blood glucose results which may have been caused by a malfunction in the measurement equipment were excluded. For quality control assurance a repeat data collection of the Nauru-STEP measures on a random sample (n = 489) of participants surveyed in 2004 was conducted in 2006. The prevalence of diabetes based on fasting blood glucose results was similar at both data collection points; 22.0% in 2004 and 22.7% in 2006 among those aged 25-64 years [8]. A total of 1,592 participants had complete data for the present analyses. There were no statistically significant differences between those excluded and those used in the analyses for age (p = 0.10), sex (p = 0.32), education (p = 0.70) or BMI (p = 0.98).

**Figure 1:**
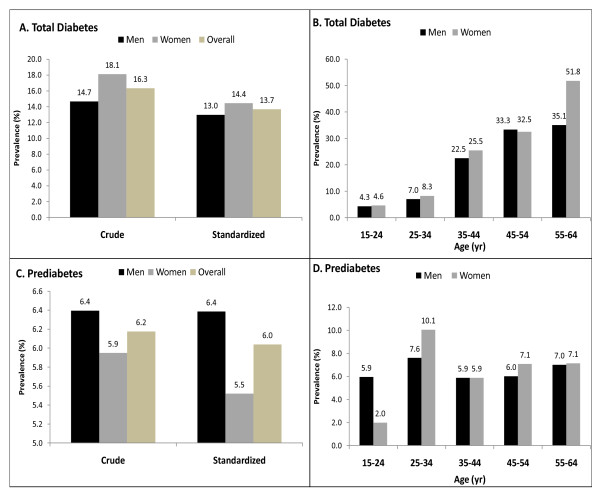
**Sex- and Age-Specific Prevalence's of Diabetes and Prediabetes among Nauruan Adults 15-64 Years of Age**. The crude and sex-standardized prevalence's of total diabetes and prediabetes are shown in Panels A and C, respectively. The crude and age-standarized prevalence's of total diabetes and prediabetes for men and women are shown in Panels B and D, respectively. Standardized prevalence's of prediabetes and diabetes is to the 2002 Nauru census. Total diabetes includes both previously diagnosed diabetes and previously undiagnosed diabetes (fasting plasma glucose ≥7 mmol/l). Prediabetes was defined as impaired fasting glucose (6.1-6.9 mmol/l).

Among those aged 15-64 years, the sex-standardized prevalence (95% CI) of diabetes and prediabetes was 13.7% (12.0, 15.4) and 6.0% (4.8, 7.3), respectively (Figure 1). Among men and women, the sex-standardized prevalence of diabetes was 13.0% (10.6, 15.4) and 14.4% (11.9, 16.9), respectively. The sex-standardized prevalence (95% CI) of prediabetes was 6.4% (4.6, 8.2) for men and 5.5% (3.9, 7.2) for women (Figure 1). The crude prevalence (95% CI) of diabetes and prediabetes for individuals aged 25 years and older were 22.0% (19.8, 24.3) and 7.1% (5.7, 8.6) respectively. The sex-standardized prevalence (95% CI) of diabetes and prediabetes for individuals aged 25 years and older was 19.9% (17.2, 22.7) and 7.3% (5.5, 9.1), respectively. Age-specific rates indicate that diabetes was more prevalent with increasing age (Figure 1). The prevalence of diabetes for individuals 15-24, 25-34, 35-44, 45-54 and 55-64 years of age was 4.5%, 7.6%, 24.1%, 32.9%, and 42.7%, respectively. The age-specific prevalence of prediabetes for the same age categories was 4.2%, 8.8%, 5.9%, 6.6%, 7.1%, respectively.

Demographic and biological characteristics of participants according to categories of fasting glucose levels (normal, prediabetes and diabetes - further classified as newly diagnosed or previously reported) found significant differences by age for both men and women (Table 1). Among men, there were significant differences by category of fasting blood glucose for age, weight, BMI, systolic blood pressure, diastolic blood pressure, cholesterol and minutes per day of physical activity. Among women, age, waist circumference, systolic blood pressure, diastolic blood pressure and cholesterol were significantly different according to fasting blood glucose levels.

**Table 1:** Study participant characteristics according to fasting glucose level in adults between 15 and 64 years of age in Nauru, 2004, by gender

	Normal	Prediabetes	New diabetes	Known diabetes	p-value trend
**MEN **(n = 800)					
N (%)	602 (78.95)	51 (6.39)	79 (8.50)	68 (6.15)	
Age in years, mean ± SE	31.21 ± 0.48	32.92 ± 1.75	39.80 ± 1.58	46.60 ± 1.18	< 0.0001
Education in years, mean ± SE	9.65 ± 0.10	9.97 ± 0.31	10.01 ± 0.24	9.90 ± 0.32	0.15
Height in cm, mean ± SE	168.20 ± 0.27	168.52 ± 0.82	168.38 ± 0.61	168.30 ± 0.61	0.78
Weight in kg, mean ± SE	91.61 ± 1.00	93.71 ± 2.95	104.77 ± 2.77	90.92 ± 2.16	0.02
BMI in kg/m^2^, mean ± SE	32.26 ± 0.32	32.97 ± 0.99	36.77 ± 0.84	32.12 ± 0.76	0.01
Waist circumference in cm, mean ± SE	100.58 ± 1.62	101.93 ± 2.33	116.55 ± 7.23	102.68 ± 1.72	0.06
Systolic blood pressure in mmHg, mean ± SE	129.49 ± 0.65	131.82 ± 2.26	134.35 ± 1.83	140.02 ± 3.13	< 0.0001
Diastolic blood pressure in mmHg, mean ± SE	77.96 ± 0.50	80.68 ± 1.59	85.02 ± 1.27	85.26 ± 1.51	< 0.0001
Heart rate in beats/minute, mean ± SE	69.08 ± 4.41	72.42 ± 2.59	81.37 ± 12.85	73.25 ± 1.19	0.44
Fasting blood glucose in mmol/l, mean ± SE	4.24 ± 0.04	6.28 ± 0.02	8.67 ± 0.27	10.41 ± 0.37	< 0.0001
Total cholesterol in mmol/l, mean ± SE	4.28 ± 0.04	4.86 ± 0.16	5.24 ± 0.16	5.18 ± 0.14	< 0.0001
High risk drinker^1^, yes %	18.57	16.82	24.82	19.72	0.63
Current smoker^1^, yes %	46.56	52.58	45.44	45.77	0.88
Fruit in serves/day, mean ± SE	0.28 ± 0.04	0.41 ± 0.14	0.24 ± 0.04	0.22 ± 0.04	0.69
Vegetables in serves/day, mean ± SE	0.48 ± 0.05	0.45 ± 0.14	0.41 ± 0.06	0.36 ± 0.05	0.31
Total physical activity in minutes per day, mean ± SE	131.49 ± 6.72	120.20 ± 24.56	121.02 ± 12.92	94.78 ± 16.50	0.11
Work physical activity in minutes per day, mean ± SE	85.67 ± 5.67	98.62 ± 23.32	89.89 ± 11.82	72.84 ± 41.19	0.81
Commuting physical activity in minutes per day, mean ± SE	24.32 ± 1.94	8.17 ± 1.89	10.58 ± 2.52	15.46 ± 4.04	0.003
Leisure physical activity in minutes per day, mean ± SE	21.44 ± 2.23	12.73 ± 5.30	20.56 ± 6.76	6.40 ± 2.42	0.06
Sedentary activity in hours per day, mean ± SE	4.16 ± 0.13	4.26 ± 0.57	4.53 ± 0.44	3.97 ± 0.42	0.81
	
	**Normal**	**Prediabetes**	**New diabetes**	**Known diabetes**	**p-value trend**
	
**WOMEN (n = 792)**					
N (%)	570 (75.93)	51 (5.95)	74 (8.49)	97 (9.62)	
Age in years, mean ± SE	32.01 ± 0.48	37.75 ± 1.59	39.62 ± 1.58	46.26 ± 0.90	< 0.0001
Education in years, mean ± SE	9.96 ± 0.10	10.11 ± 0.42	10.20 ± 0.23	9.96 ± 0.20	0.63
Height in cm, mean ± SE	156.93 ± 0.22	156.31 ± 0.72	157.11 ± 0.60	156.56 ± 0.65	0.65
Weight in kg, mean ± SE	83.15 ± 0.92	82.02 ± 2.72	91.10 ± 2.46	83.73 ± 1.93	0.13
BMI in kg/m^2^, mean ± SE	33.68 ± 0.36	33.50 ± 1.03	36.86 ± 0.95	34.03 ± 0.69	0.09
Waist circumference in cm, mean ± SE	96.16 ± 0.70	96.89 ± 2.22	105.10 ± 1.94	100.77 ± 1.42	< 0.0001
Systolic blood pressure in mmHg, mean ± SE	118.91 ± 0.67	123.29 ± 3.19	125.34 ± 2.32	215.34 ± 2.24	0.0001
Diastolic blood pressure in mmHg, mean ± SE	76.55 ± 0.46	78.33 ± 1.80	79.33 ± 1.22	78.32 ± 1.15	0.03
Heart rate in beats/minute, mean ± SE	75.99 ± 1.92	71.64 ± 1.42	74.22 ± 1.95	76.06 ± 1.03	0.82
Fasting blood glucose in mmol/l, mean ± SE	4.28 ± 0.04	6.29 ± 0.02	8.71 ± 0.22	10.88 ± 0.27	< 0.0001
Total cholesterol in mmol/l, mean ± SE	4.54 ± 0.04	5.65 ± 0.23	5.15 ± 0.16	5.60 ± 0.14	< 0.0001
High risk drinker^1^, yes %	10.62	12.43	10.63	12.63	0.94
Current smoker^1^, yes %	56.37	60.54	51.81	59.37	0.77
Fruit in serves/day, mean ± SE	0.26 ± 0.03	0.14 ± 0.03	0.27 ± 0.05	0.28 ± 0.08	0.92
Vegetables in serves/day, mean ± SE	0.58 ± 0.05	0.52 ± 0.13	0.64 ± 0.13	0.61 ± 0.15	0.79
Total physical activity in minutes per day, mean ± SE	80.44 ± 5.17	85.76 ± 18.41	123.92 ± 18.03	79.15 ± 15.21	0.25
Work physical activity in minutes per day, mean ± SE	56.67 ± 4.86	67.47 ± 17.85	89.65 ± 15.42	57.55 ± 14.89	0.29
Commuting physical activity in minutes per day, mean ± SE	18.93 ± 1.54	11.35 ± 3.17	18.67 ± 4.05	16.63 ± 3.79	0.50
Leisure physical activity in minutes per day, mean ± SE	5.19 ± 1.12	4.97 ± 2.88	15.09 ± 7.65	4.13 ± 1.59	0.38
Sedentary activity in hours per day, mean ± SE	3.74 ± 0.13	3.14 ± 0.45	3.50 ± 0.41	3.60 ± 0.36	0.47

^1^High risk drinkers included respondents who reported consuming 3 to 9 drinks per day on 5 or more days a week in the past 12 months. Tobacco use was assessed according to participants' response to whether they currently smoke any tobacco products such as cigarettes, cigars or pipes (yes or no). Physical activity was measured using the Global Physical Activity Questionnaire (GPQAQ), which measures the frequency and duration of physical activity in work, travel and leisure domains [24,25].

Undiagnosed diabetes was present in approximately 9% of men and women and was significantly more prevalent among those who were older (mean ± age: 39.8 years ± 1.6 vs 46.6 ± 1.2, p < 0.0001 for men and 39.6 ± 1.6 vs. 46.3 ± 0.9, p < 0.0001 for women,) and had greater BMI values (36.8 ± 0.8 vs. 32.1 ± 0.8, p = 0.01 for men and 36.9 ± 1.0 vs. 34.0 ± 0.7, p = 0.09 for women) compared to those with known diabetes (Table 1). Compared to men with normal fasting plasma glucose, prediabetic and diabetic men showed a significantly higher age-adjusted prevalence of high cholesterol and high BMI (Table 2). Compared to women with normal fasting plasma glucose, prediabetic and diabetic women had higher diastolic blood pressure, cholesterol levels and waist circumference measurements (Table 2).

**Table 2:** Adjusted odds ratio (OR) and 95% CIs for diabetes and prediabetes compared with normal glucose, as determined by polychotomous multiple logistic regression analysis using the generalized logit model

	Prediabetes		Diabetes	
	
Variables	OR (95% CI)	p-value	OR (95% CI)	p-value
Age	1.00 (0.98, 1.03)	0.77	1.06 (1.04, 1.07)	**< 0.0001**
Sex				
Male	1.00	0.75	1.00	0.17
Female	0.92 (0.54, 1.55)		1.78 (0.55, 1.12)	
Education	1.04 (0.94, 1.16)	0.47	1.00 (0.93, 1.08)	0.93
BMI (per 5 kg/m^2^)	0.92 (0.85, 0.99)	**0.02**	0.94 (0.90, 0.99)	**0.02**
Waist circumference (cm)	1.04 (1.00, 1.08)	**0.05**	1.04 (1.02, 1.07)	**0.002**
Systolic blood pressure (mmHg)	1.00 (0.98, 1.02)	0.75	1.00 (0.98, 1.01)	0.58
Diastolic blood pressure (mmHg)	1.01 (0.98, 1.04)	0.54	1.00 (0.98, 1.02)	0.71
Heart rate (beats/minute)	0.99 (0.97, 1.01)	0.27	1.00 (0.99, 1.01)	0.51
Total cholesterol (mmol/l)	2.02 (1.66, 2.47)	**< 0.0001**	1.84 (1.58, 2.14)	**< 0.0001**
High risk alcohol consumption				
No	1.00	0.68	1.00	0.41
Yes	0.87 (0.44, 1.70)		1.20 (0.78, 1.85)	
Current smoker				
No	1.00	0.45	1.00	0.49
Yes	1.20 (0.74, 1.94)		0.89 (0.65, 1.23)	
Fruit (serves/day)	1.09 (0.81, 1.47)	0.56	0.98 (0.78, 1.23)	0.87
Vegetables (serves/day)	0.91 (0.72, 1.16)	0.45	0.92 (0.79, 1.08)	0.30
Overall physical activity (minutes per day)	1.00 (0.99, 1.00)	0.50	1.00 (0.99, 1.00)	0.86
Sedentary activity (hours per day)	0.95 (0.88, 1.03)	0.21	0.98 (0.92, 1.03)	0.34

In the multivariable, multinomial, logit models, risk of prediabetes increased with increasing cholesterol levels (OR: 2.02, 95% CI: 1.66, 2.47) and waist circumference (OR: 1.04, 95% CI: 1.00, 1.08). Diabetes risk increased with increasing cholesterol levels (OR: 1.84, 95% CI: 1.58, 2.14) and waist circumference (OR: 1.04, 95% CI: 1.02, 1.07). Every year increase in age was associated with a 6% increase in the risk of diabetes compared to normal fasting glucose levels (95% CI: 1.04, 1.07) (Table 3). BMI was inversely associated with risk of prediabetes (OR: 0.92, 95% CI: 0.85, 0.99) and diabetes (OR: 0.94, 95% CI: 0.90, 0.99).

**Table 3:** Age-adjusted prevalence for cardiovascular risk profile according to fasting glucose level

	Normal	Prediabetes	Diabetes*	p value
**Men**				
Elevated systolic blood pressure (≥140 mmHg)*	152.1 ± 1.2	152.9 ± 2.7	150.4 ± 1.4	0.56
Elevated diastolic blood pressure (≥90 mmHg)	97.6 ± 0.8	95.4 ± 0.9	96.3 ± 0.5	0.16
High total cholesterol (≥6.21 mmol/l; ≥240 mg/dl)	6.8 ± 0.2	8.9 ± 0.0	7.6 ± 0.2	< 0.0001
BMI ≥ 25 kg/m^2^	33.9 ± 0.3	33.4 ± 0.8	36.3 ± 0.70	0.005
Waist circumference	108.9 ± 1.8	105.7 ± 1.7	110.1 ± 3.1	0.29
Minutes per day of physical activity (low status)	122.5 ± 5.9	104.2 ± 20.2	121.3 ± 14.3	0.69
**Women**				
Elevated systolic blood pressure (≥140 mmHg)	156.2 ± 2.9	162.8 ± 4.3	152.7 ± 2.8	0.15
Elevated diastolic blood pressure (≥90 mmHg)	96.4 ± 1.0	99.0 ± 1.9	93.4 ± 0.4	0.0006
High total cholesterol (≥240 mg/dl)	6.9 ± 0.1	8.5 ± 0.2	7.6 ± 0.9	< 0.0001
BMI ≥ 25 kg/m2	34.7 ± 0.3	35.9 ± 0.6	36.3 ± 1.3	0.13
Waist circumference	99.2 ± 0.6	97.9 ± 3.0	107.3 ± 1.3	< 0.0001
Minutes per day of physical activity (low status)	72.7 ± 4.3	64.8 ± 13.9	86.1 ± 15.3	0.58

Data are means ± SEM. Age-adjusted prevalence's were standardized using the national census in 2002 from the Nauru National Bureau of Statistics Office. Waist Circumference: > 90 cm for men and > 80 cm for women. Low physical activity status: < 600 MET minutes per week. *P *values were calculated using a linear contrast test.

* Diabetes includes both previously diagnosed diabetes and previously undiagnosed diabetes.

**Figure 2:**
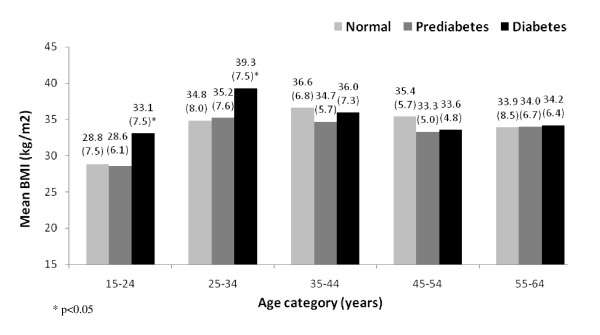
**Mean BMI (± SD) for respondents with normal, prediabetes and diabetes according to age categories**. Mean BMI (± SD) values for respondents with normal, prediabetes and diabetes status by age categories. Diabetes includes both previously diagnosed diabetes and previously undiagnosed diabetes (fasting plasma glucose ≥7 mmol/l). Prediabetes was defined as impaired fasting glucose (6.1-6.9 mmol/l).

To further examine the relationship between BMI and risk of prediabetes and diabetes, a number of additional analyses were performed. Mean BMI (± SD) values were examined across narrowly defined age categories (Figure 2). Unadjusted significance testing found significantly higher mean BMI values among diabetics aged 15-24 years old (OR: 1.10, 95% CI: 1.04, 1.17, p = 0.0019) and 25-34 years old (OR: 1.07, 95% CI: 1,02, 1.17, p = 0.004) compared to those with normal fasting glucose concentrations. No other significant associations between BMI and prediabetes or diabetes by age group were found.

In the multivariable, multinomial, logit model, the interaction between age and BMI was not significant for prediabetes (p = 0.96) or diabetes (p = 0.07). In a multivariable, multinomial, logit model with age as an ordinal variable (15-24, 25-34, 35-44, 45-54 and 55-64 years) no significant association between any of the age categories and risk of prediabetes was found. Risk of diabetes was significantly higher among those 35-44 years old (p < 0.0001), 45-54 years old (p < 0.0001) and 55-64 years old (p < 0.0001), with no other mentioned differences compared to findings presented in Table 3.

In a multivariable, multinomial, logit model which examined variables according to standardized cutoffs, including overweight (≥25 kg/m2), obesity (≥30 kg/m2), increase waist circumference risk (≥90 cm for men and ≥80 cm for women), high systolic blood pressure (≥130 mmHg), diastolic blood pressure (≥85 mmHg), high cholesterol (≥5.21 mmol/L) and low physical activity, BMI categorized as normal, overweight, or obese was no longer significantly associated with prediabetes or diabetes. Age, waist circumference, and cholesterol remained significant and were similar to findings reported in Table 3.

## Discussion

This study presents the most recent published age-specific and sex-standardized prevalence estimates for diabetes and prediabetes among Nauruans aged 15-64 years since a series of surveys conducted in 1975, 1976, 1982 and 1987 [1,3-5]. The present study found a sex-standardized diabetes prevalence (95% CI) of 13.7% (12.0, 15.4). A similar prevalence was found in a random sample of participants from the current study (2004 Nauru-STEPS survey) followed up in 2006 [8]. Among those aged 25 years, the crude prevalence of diabetes in 2004 was 22.0% (95% CI: 19.8, 24.3) compared to 22.7% (95% CI: 18.7, 26.7) in 2006 [8].

In this study, diabetes rates were significantly higher among women compared to men (14.4% vs. 13.0%) and showed age cohort differences, with higher rates of diabetes presenting in older age groups. The prediabetes prevalence was relatively similar across age groups, with the exception of markedly higher prediabetes prevalence among individuals aged 25-34 years old. An increase in diabetes prevalence with increasing age is similar to previous findings from Nauru [1].

Thirty years ago, results from Nauru found that there were two new diabetics for each known diabetic [1]. In comparison, results from this study found there was one new diabetic for each known diabetic. More than half (52.0%) of those with diabetes were previously undiagnosed. Individuals with undiagnosed diabetes had significantly higher BMIs and were significantly younger than those with known diabetes. The age range of unknown diabetics ranged from 26 to 65 years indicating early detection and broader population screening is needed, starting as young as 30 years of age. Moreover, the fact that diabetes affects people of reproductive age (20 s and 30 s) in Nauru carries important public health implications for pregnant women and their infants [11].

While diabetes remains a major public health problem in Nauru, this study indicates that the diabetes prevalence in Nauru has declined compared to two decades ago. These results confirm previous research which found diabetes prevalence in Nauru has declined over time [5]. The diabetes prevalence in Nauru was reported as 34.4% in 1975^1^, 29.0% in 1976,^3 ^23.9% in 1982^4 ^and 24.0% in 1987 [12]. The decline in the prevalence observed in these cross-sectional surveys is consistent with the results of a longitudinal analysis of a cohort of Nauruans, which found that the age-standardized prevalence (95% CI) of NIDDM decreased from 27.9% (23.8, 32.0) in 1975/6 to 24.7% (22.7, 26.6) in 1982 to 24.0% (22.1, 25.9%) in 1987 [5].

The specific reasons for the decline are not certain. Evidence from previous studies support a strong genetic component for diabetes among Micronesians in Nauru [6,13]. Early data pointed to NIDDM clustering in certain ethnic groups, especially the Pima Indians, Nauruans and Australian Aborigines, lending some support to the thrifty gene hypothesis [14]. The thrifty gene hypothesis postulates that metabolically thrifty genes allow more efficient food utilization, fat deposition and rapid weight gain at occasional times of food abundance, for better survival during a subsequent famine [6].

In 1991, Dowse and colleagues reported a decline in the incidence of abnormal glucose tolerance in Nauru, which they attributed to the fall in the population frequency of the thrifty genotype due to higher mortality and lower fertility among diabetic Nauruans [5]. However, work by Barker and colleagues suggested that the size and speed of change was unlikely a eugenic effect of low reproduction among diabetics and more likely a result of improved fetal and infant nutrition in the postwar generation [11]. Counter arguments of the thrifty gene hypothesis warn against using this to explain high rates of chronic disease in post-colonial indigenous societies because it confuses "genes" with "race", and does not give due credit to epidemiological data that shows the effects of social class, education, income, poverty, ethnicity and prior malnutrition on chronic diseases [15].

The 20-fold increase [3] in diabetes prevalence from the 1960s to the 1980s has largely been attributed to lifestyle changes of Nauruans, especially a shift from a healthier and more traditional diet to consuming imported processed foods during a period of extreme wealth.^1 ^The prevalence of diabetes in Nauru during the 1970s was alarmingly high and represented one of the highest rates in the world [14,16]. Over the past decade the economic state of Nauru has changed drastically; the phosphate industry collapsed, leading Nauru to suffer a dramatic decline in its economy, and becoming more dependent on foreign aid [8]. The decline in diabetes prevalence in Nauru may be partly attributed to the rapid change in Nauru from an excessively wealthy state to that of a poor state. However, evidence to support this explanation is lacking. None of the dietary or physical activity measures in this study or in fact, in previous surveys [5,16], were significantly associated with increased risk of prediabetes or diabetes compared to those with normal levels.

The lack of effects from modifiable risk factors for diabetes in the present study may be due to limited data on these risk factors or limited variation in the data collected. Aside from fruit and vegetable intake, there were no other dietary variables collected in this study. Notably there was evidence of increased waist circumference and cholesterol levels among prediabetics and diabetics suggesting that excess body fat and dietary fat intake are important risk factors for diabetes and cardiovascular disease in this population. Data on physical activity was comprehensively measured in this study; however, levels of all types of physical activity during work, leisure and transport were extremely low regardless of diabetes status. Only 1.2% of Nauruans met the Global Physical Activity Questionnaire health-enhancing cut-off for moderate to high levels of overall physical activity [8]. The incredibly high rate of physical *inactivity *in Nauru appears to be greater than most, if not all countries, worldwide. Results from a 2007 study of 51 countries found the age-standardized country prevalence of physical inactivity ranged from 1.6% (Comoros) to 51.7% (Mauritania) for men, and from 3.8% (Comoros) to 71.2% (Mauritania) for women [17].

The finding that BMI had a significant protective effect on diabetes when examined as a continuous variable is an interesting one. Detailed analyses suggest the relationship between BMI and diabetes is confounded by age, although the interaction term was not statistically significant. Lack of significance may be a result of insufficient sample size and a disproportionately younger distribution in the population. Another explanation for the possible protective effect of BMI on diabetes is that BMI is affected by overall body structure and size, and in Pacific Islanders, central or upper-body fat distribution (as assessed by waist-hip ratio, circumferences, or skinfolds) may be more relevant in terms of increased risk of obesity-related health problems [18]. In the present study, waist circumference was consistently a significant risk factor for prediabetes and diabetes. Another possibility for why BMI has a protective effect on diabetes is that obesity in Nauru may be associated with a survival advantage. A study on mortality in Nauru failed to find an increase in total or cardiovascular disease mortality at body mass indices above 35 kg/m^2 ^[18]. As suggested by Hodge et al, older diabetic subjects may be leaner because of poorly controlled diabetes resulting in weight loss associated with energy loss as glycosuria rather than a positive effect of treatment [18].

Differences in glucose measurement and sampling methodology affect comparison of the present study to previous surveys in Nauru. The present study relies on fasting plasma glucose concentrations and includes a nationwide representative sample of Nauruan adults aged 15 years and older [8]. In contrast, the 1975 survey collected information on Nauruans aged 15 years and older residing in two of the island's fourteen districts and defined diabetes as a 2-hour post-loading plasma glucose of 160 mg/100 ml (~8.9 mmol/l) or more [1]. In 1976, individuals aged 10 years and older were tested at a local hospital after responding to local media announcements and diabetes was defined as a 2-hour post-loading plasma glucose of 160 mg/100 ml or more [3]. In 1982, a population survey was conducted among persons aged 20 years and older and diabetes was defined as a 2-hour post-loading plasma glucose of 200 mg/100 ml or more, or in the absence of a 2-hour specimen, a fasting plasma glucose level of 140 mg/100 ml (7.8 mmol/L) [4]. In 1987, all individuals aged 15 years and over living on the island had a random or fasting plasma glucose determined and those who had participated in 1975/76 and/or 1982 surveys and had not been previously determined to be diabetic had an oral glucose tolerance test taken [5].

While diabetes prevalence has decreased in Nauru over the last thirty years, results from this study indicate that the rate of diabetes is still very high. Similar or higher rates of diabetes have been reported in other Micronesian populations in the Pacific region [19,20]. For example, the prevalence of diabetes (fasting blood glucose ≥ 6.1 mmol/L) in Kiribati individuals aged 22-64 years old was 28.1% in 2006 [20] and in Pohnpei, the prevalence of diabetes (fasting blood glucose ≥ 7.0 mmol/L) among individuals aged 25-64 year olds was 32.1% in 2002 [19]. In other parts of the world, the prevalence of diabetes is notably lower. For instance, the sex standardized prevalence of total diabetes in the United States in 1999-2002 was 6.5% (95% CI: 5.9-7.2) among individuals 20 years and older [21] and 9.7% (95% CI: 9.2-10.1) in China among adults 20 years of age or older [22].

Strengths of this study include a large population-based sample, representative sampling methodology and the use of standardized data collection protocols. The survey had a high response rate (89.7%) and examined 15-20% of island's total population enabling nationwide estimates of chronic disease-related information. A recognized limitation in the present study is the use of a fasting plasma glucose concentration for defining diabetes. The predictive value of fasting plasma glucose for the diagnosis of diabetes has been found to improve in higher prevalence groups; however, it has also been found to have lower sensitivity than the oral glucose tolerance tests (OGTT) among Micronesians [23]. Other study limitations include the use self-reported measures for behavioral risk factors, and possible biases from incomplete data due to non-respondents and missing item response data.

## Conclusion

Overall, this study demonstrates that while the prevalence of diabetes in Nauru is lower than it was last reported to be twenty years ago, diabetes remains a major problem in Nauru and requires ongoing public health action.

## Competing interests

The authors declare that they have no competing interests.

## Authors' contributions

AK wrote the manuscript and analyzed the data; PP designed, co-ordinated and managed in-country data collection, contributed to the discussion and reviewed/edited the manuscript; BS designed and managed in-country data collection, quality checked data and reviewed/edited the manuscript; KK reviewed/edited the manuscript and facilitated in-country data collection; LD facilitated 2006 survey and reviewed/edited the manuscript; AF managed 2006 biochemical data collection and analyses, reviewed/edited the manuscript; AB reviewed/edited the manuscript. All authors read and approved the final manuscript.

## Pre-publication history

The pre-publication history for this paper can be accessed here:

http://www.biomedcentral.com/1471-2458/11/719/prepub
